# Low Fraction Size Re-irradiation for Large Volume Recurrence of Glial Tumours

**DOI:** 10.1007/s12253-020-00868-2

**Published:** 2020-07-09

**Authors:** Ágnes Dobi, Barbara Darázs, Emese Fodor, Adrienne Cserháti, Zsófia Együd, Anikó Maráz, Szilvia László, Leopold Dodd, Zita Reisz, Pál Barzó, Judit Oláh, Katalin Hideghéty

**Affiliations:** 1 Department of Oncotherapy, University of Szeged, Korányi fasor 12, Szeged, H-6720 Hungary; 2 Department of Pathology, University of Szeged, Állomás utca 1, Szeged, H-6725 Hungary; 3 Department of Neurosurgery, University of Szeged, Semmelweis utca 6, Szeged, H-6725 Hungary

**Keywords:** Re-irradiation, Multiform glioblastoma, Glioma

## Abstract

The aim of the present study was to evaluate the efficacy of re-irradiation (re-RT) in patients with advanced local relapses of glial tumours and to define the factors influencing the result of the hyper-fractionated external beam therapy on progression after primary management. We have analysed the data of 55 patients with brain tumours (GBM: 28) on progression, who were re-irradiated between January 2007 and December 2018. The mean volume of the recurrent tumour was 118 cm^3^, and the mean planning target volume (PTV) was 316 cm^3^, to which 32 Gy was delivered in 20 fractions at least 7.7 months after the first radiotherapy, using 3D conformal radiotherapy (CRT) or intensity modulated radiotherapy (IMRT). The median overall survival (mOS) from the re-RT was 8.4 months, and the 6-month and the 12-month OS rate was 64% and 31%, respectively. The most important factors by univariate analysis, which significantly improved the outcome of re-RT were the longer time interval between the diagnosis and second radiotherapy (*p* = 0.029), the lower histology grade (*p* = 0.034), volume of the recurrent tumour (*p* = 0.006) and Karnofsky performance status (KPS) (*p* = 0.009) at the re-irradiation. Our low fraction size re-irradiation ≥ 8 months after the first radiotherapy proved to be safe and beneficial for patients with large volume recurrent glial tumours.

## 1. Introduction

Gliomas, with incidence of 5/100 000 in adults, are the most common primary central nervous system malignancies, peaking between the fifth and sixth decades of life [1]. After initial multimodal treatment, at least 70% recurrence rate of gliomas can be expected [2–4]. By surgical therapy alone, the disease has a very poor prognosis (median survival 4–6 months [5], whereas surgery accompanied by radiotherapy (RT) ameliorates the median survival data to 8–9 months. Together with concomitant and sequential TMZ, better median survival values can be expected, such as 15 months for glioblastomas, or even 2–5 years for anaplastic gliomas [6].

In the case of recurrence with its considerable limitations, and only if it is possible, surgical treatment has the highest efficacy [7]. In certain good performance status patients with good anatomical access to tumours, surgery is applicable, but the resection outcome could be definitely limited by considerable infiltration of nervous tissue and by higher morbidity risk [8, 9]. As for other low grade and grade 3 cases, temozolamide (TMZ) is the treatment of choice, if it was not administered during the initial management. Thereafter and for GBM second-line systemic treatment (such as chemo- or biological therapy) and re-irradiation is optional, in the lack of standardised treatment for recurrent gliomas [10]. Recently, Tumour Treating Field (TTF), a novel therapeutic option emerged prolonging the survival with further 6 months [1]. For systemic treatment, monoclonal antibody (bevacizumab), chemotherapy (nitrosurea, lomustine, dose dense TMZ [9], immune checkpoint blockade (nivolumab, pembrolizumab) [11], or even vaccines (DCVax) [12–14] are options to consider. For recurrent tumours, salvage re-irradiation could be selected. The typical re-irradiation techniques and strategies for recurrent gliomas are conventionally fractionated RT, brachytherapy, hypofractionated stereotactic radiosurgery (FSRT), stereotactic radiosurgery (SRS) alone, or combination treatment with RT and systemic therapy, and palliative RT [15, 16].

On reviewing several clinical trials, the 6- and 12-month overall survival (OS-6 and OS-12), calculated from the time of re-irradiation, were 73% and 36%, respectively, whereas the 6- and 12-month of progression free survival (PFS-6 and PFS-12) were 43% and 17%, respectively [17]. Median OS (mOS) was 7.4–12.7 months in other studies [18–22].

## 2. Materials and Methods

Between 2007 and 2018, at the Department of Oncotherapy, altogether 55 patients with recurrent glial tumours were subjected to re-irradiation. The present study has been carried out in accordance with The Code of Ethics of the World Medical Association (Declaration of Helsinki) for experiments involving humans. Informed consent was obtained from the patients at their first clinical admission for the anonymised use of their patient data for research purposes. According to Sect. 20/Q of No. 23/2002 Decree of the Ministry of Health, Hungary, the present study is considered as a non-interventional clinical study. The whole present study was carried out according to the ethical permission No. 4209/2018-SZTE, issued by the Ethical Committee of our University. The treatment schemes were thoroughly discussed with every single patient, independently from their actual performance status. The re-irradiation was agreed by signed informed consent. The initial care consisted of surgery in each case. The patients with grade 2 and grade 3 brain tumours received radiotherapy only postoperatively and for GBM we applied adjuvant chemoradiation therapy followed by temozolamide monotherapy up to progression. Magnetic resonance imaging (MRI) were performed three monthly. Disease progression was defined by two independent experts. At the time of diagnosis, the tumour grading was based on histological assessment. At the time of re-RT, histological evaluation was performed only in the re-operated cases, in the case of the remaining patients (without re-operation), the grading was based on clinical and radiological evaluation. The re-irradiation volume was defined on the basis of planning CT (computed tomography) and MRI fusion. Patients were immobilised with a 3-point thermoplastic mask (ORFIT Industries, NL). The planning target volume encompassed the GTV (gross tumour volume) plus 0.3-1 cm margin. The shapes of the recurrent tumours were frequently highly irregular, sometimes with multiple manifestations, and with spread to the contralateral hemisphere through the corpus callosum; or spreading along the wall of the previous surgical cavity and/or ventricle wall, resulting in larger PTV (planning target volume). The normal structures were contoured including the lens, optic chiasm, optic nerve, brain, and brainstem. Treatment planning was performed with Eclipse (version 5, Varian Medical Systems, Palo Alto, USA). The re-RT dose was 32 Gy in 1.6 Gy daily fractions in all cases, in order to avoid serious neurotoxicity. Dependent on the location and extent of the recurrent glioma, 3 DCRT or IMRT or VMAT (Rapid Arch) therapy-plans (VMAT) were generated according to the ICRU (International Commission on Radiation Units & Measurements, Inc.) 52 recommendation. [23].

During brain irradiation, patients received 12 mg methyl-prednisolone for prevention of brain oedema, with gradually decreased dosing after radiotherapy. The dose of methyl-prednisolone was adjusted according to the symptoms of intracranial pressure elevation due to brain oedema. The majority of the patients (32 over 23) received bevacizumab therapy after the re-RT, and these patients were controlled in a bi-weekly fashion, with physical examination up to progression and 3-months intervals MRIs were performed, whereas for the remaining group without bevacizumab treatment after re-RT, the check-ups were scheduled in 4–6 weeks. Two experts evaluated the images according to the RANO HGG (Response Assessment in Neuro-Oncology High-grade glioma) criteria [24]. We included all patients with a recurrent glial tumour who completed the 32 Gy re-irradiation in 20 fractions to the present analysis. We assessed retrospectively the overall survival (OS) from the diagnosis, and from the first day of the re-irradiation according to the, age, Karnofsky performance score (KPS), primary tumour grade and histopathology type, the type of the primary tumour removal, size of GTV, size of PTV, time interval between two irradiations, time elapsed between diagnosis and 2nd RT, second line bevacizumab treatment. The data were evaluated by Kaplan-Meier statistical analysis with IBM SPSS Statistics for Windows, Version 20.0 (Armonk, NY: IBM Corp.) p value < 0.05 was considered as statistically significant. COX regression was used for univariate, as well as multivariate analysis. Factors with significance in univariate analysis were included into a multivariate analysis. After the re-irradiation we recorded the KPS, Mini Mental Score (MMS) and daily activity in every visit.

## 3. Results

### 3.1 Patient Characteristics

*Table *1* summarises the patient characteristics.* The mean age of the population at the time of the primary diagnosis detection was 39 years (range: 11–71 years); 49% of them was male and 51% female. The mean age at the time of the re-irradiation was 42 years (range: 13–72 years). The KPS was in 40% of this population over 70%. At the beginning of the reirradiation, majority of the patients had minor neurological symptoms, such as hemiparesis, facial paresis, focal seizure partly controlled by antiepileptic medication, more frequently motor and sensory aphasia. Besides these symptoms, the patients preserved the ability of self-caring, except 5 patients with serious paresis needing regular help in their daily life. Out of 23 cases with repeated surgery, only four initially grade 2–3 tumours showed malignant transformation to grade 3–4. In the majority of the cases, though, reoperation took place relatively early during the course of the disease. Usually in the case of initially low grade tumours, surgery was performed prior to the first oncological management; and also grade 3 tumours were as well as re-operated some years prior to reirradiation. In 84% of the cases, based on the clinical behaviour of the tumour, their malignant transformation was highly probable at the time of reirradiation, but no regular biopsy was performed in order to confirm it. The average time interval between the diagnosis and re-irradiation was 47.4 months (range: 7.3–228 months) first and the re-irradiation was 36 months (range: 7.7–232 months) respectively. All patients received first-line systemic temozolomide treatment, either as part of initial postoperative management (GBM), or at the first relapse. 23 patients were treated with bevacizumab monotherapy, as second-line treatment. The re-irradiation was performed after the first-line systemic treatment in 45 cases, and after second-line therapy in 10 cases. The primary histological type was grade 2 astrocytoma in 15 cases, grade 3 glial tumour (anaplastic astrocytoma or oligodendroglioma) in 12 cases, and glioblastoma multiforme (GBM) in 28 cases. MGMT (O-6-methylguanine-DNA methyltransferase) methylation status was known in 26 cases. 16 patients were methylated, 9 patients borderline methylated and in one case MGMT was non-methylated.

**Table 1:** Summary of the patient characteristics

Variables	No. of the patients
Number of the patients	55
Sex	
Male	27
Female	28
KPS	
>70%	22
≤ 70%	33
Primary histopathology type	
astrocytoma grade 2	15
oligodendroglioma grade 3	6
anaplastic astrocytoma grade 3	6
glioblastoma multiforme	28
Salvage surgery	23
Prior temozolomid treatment	55
MGMT methylation status	
methylated	18
unmethylated	9
unknown	28

### 3.2 Survival Analysis

Median survival was altogether 42.6 months, as calculated from the date of the first diagnosis. *The Table *2* shows the survival data.* Regarding histology, cases with lower, grade 2 malignancies had the most favourable survival values (111.0 months), whereas this value was 23 months (p < 0.001) in cases with GBM. We found a strong correlation to histological type: grade 2 astrocytoma cases had the longest survival (114.8 months), whereas the worst survival was detected of grade 4 cases (30.7 months; p ≤ 0.001).

**Table 2:** Survival data. Significant correlations between investigated factors are highlighted with bold characters

Variable	n	OS (months)	± SE	p-value
		from initial diagnosis		
Entire group	55	42.6	2.6	
initial histopathology type				
grade 2	15	114.8	40.2	**p < 0.001**
grade 3	12	52.2	9.8	
grade 4	28	30.7	1.3	
		from re-RT		
entire group	55	8.37	1.9	
histopathology type at re-irradiation				
grade2 (n = 12) + grade3 (n = 14)	26	10	1.2	**p = 0.031**
grade 4	29	6	2	
GTV re-RT mean 118 cm^3^				
≤ mean	29	12.9	3.9	**p = 0.006**
> mean	23	5.5	0.3	
KPS at re-RT				
≤70%	33	5.6	0.7	**p = 0.009**
>70%	22	10.4	1.9	
Time between diagnosis (DG) and re-RT				
≤47 months	18	6.7		**p = 0.029**
>47 months	37	10.2		
PTV re-RT 316 cm^3^				
≤ mean	33	10.1	1.5	p = 0.246
> mean	22	5.5	0.4	
Age at re-irradiation				
≤40 year	27	8.3	2.2	p = 0.704
>40 year	28	6.6	2.7	
bevacizumab therapy before re-RT				
no	32	6.5	1.1	p = 0.35
yes	23	10.2	0.3	

The most important factors significantly influencing the outcome of re-RT were the time interval between the first and second radiotherapy, histology grade, GTV, and KPS at the re-irradiation.

### 3.3 Survival from the Beginning of Re-irradiation

The mOS from the re-RT of the entire cohort was 8.4 months; 6 patients survived more than 10 months and 2 patients more than 2 years. The 6-month and the 12-month OS rate was 64% and 31% respectively.

**Fig. 1:**
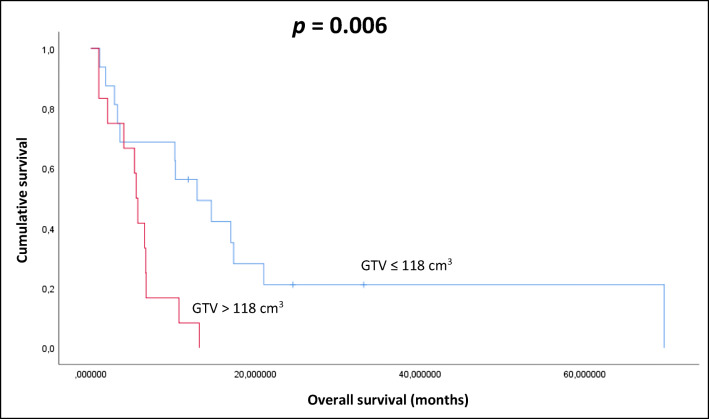
Kaplan-Meier plot of the correlation between GTV-re-irradiation and OS (p=0.006)

The mean volume of GTV, as contoured during Re-RT, was 118.0 cm^3^ (range: 4.5–304 cm^3^). Patients with lesser than average GTV at re-RT had 12.9 months, patients with greater than average GTV at re-RT had 5.5 month of median survival (p = 0.006) (Fig. 1).

**Fig. 2:**
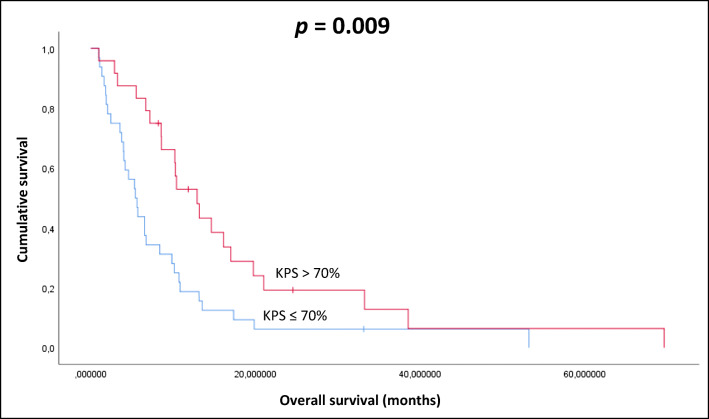
Kaplan-Meier plot of the correlation between KPS and OS (p=0.009)

Patients with KPS > 70% at the beginning of re-RT had significantly better survival values (10.4 months, p = 0.009), than those ones with poorer general conditions (5.6 months) (Fig. 2).

**Fig. 3:**
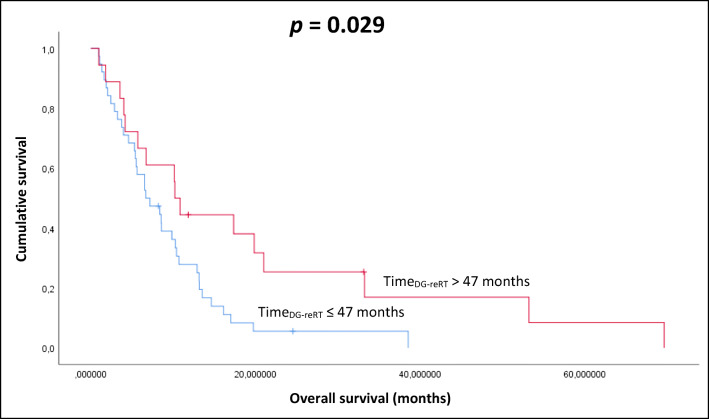
Kaplan-Meier plot of the correlation between time (DG-reRT) and OS (DG=diagnosis; p=0.029)

Comparing time interval (between diagnosis and second radiotherapy, reRT) with OS on univariate analysis, patients with an interval of more than 47 months from 1st to the 2nd course of RT (mOS 10.2 vs. 6.7 months, hazard ratio (HR) 0.99, 95% confidence interval (CI) p = 0.029. (Fig. 3).

Median survival, as calculated from re-irradiation for the entire group was 9.0 months. According to histopathology GBM: 6.0 months, grade 2 + 3 malignancies 10.0 months; p = 0.031.

These factors proved to be significant for OS from re-RT in cox-regression univariate analysis. In multivariate analysis, the smaller GTV and better KPS remained significant influencing factors.

In the present study, no significant interrelation was found between OS and age at re-RT, size of PTV, type of primary surgical intervention, or whether the second-line bevacizumab therapy preceded or followed the re-irradiation.

### 3.4 Outcome and Toxicity of the Treatment

Due to the second radiotherapy at progression, amelioration of neurological signs and KPS were experienced in 58% of our patients. Control radiological imaging detected stable disease or partial remission in 44 cases.

In the majority of the cases, the prophylactic dose and escalated dose on demand of the methyl-prednisolone prevented the development of serious brain oedema and the consequent intracranial pressure elevation. We did not see any radiation necrosis on the follow up MRIs and no major cognitive deficit was associated to the re-RT assessed with MMS.

## 4. Discussion

In the past, re-irradiation for recurrent malignant gliomas was considered with great reluctance due to the high risk of radiation necrosis. In the recent decades after implementation of advanced RT techniques, several retrospective analyses, reviews, as well a prospective study and meta-analysis were published proving that re-irradiation is useful treatment option for recurrent brain tumours [16, 17, 25, 26].

The present work represents the establishment of a careful, consequent re-RT approach with low fraction size to avoid radiation sequelae using standardised target volume and dose definition even for large volume recurrences. Selected studies on SRS for small volume recurrences of median 6.2 to 28.0 cm^3^ [27–29] showed an improvement on median survival ranging from 5.3 to 13.0 months with associated radionecrosis of 0–31.3%. Others have reported their results for recurrent GBM volumes of median 7–50 cm^3^ applying FSRT resulted in median survival within the range of 6.5–11 months [26, 30–32]. In contrast to these small target volumes for re-RT with SRS techniques, lower doses to larger volumes could be applied safely with acceptable efficacy, which was confirmed by the first meta-analysis on re-irradiation published by Kazmi et al. [17]. However, highly divergent fractionation schemes and target volume concepts are applied with heterogeneous median survival times between 5 and 18 months. Krauze et al. reported a mOS of 6 months after re-irradiation of recurrent glioma with median 30 Gy [33]. Another recent study revealed that OS after salvage SRS or hypofractionated RT (HFRT) does not significantly (p = 0.06) differs from that after conventionally fractionated re-RT, and the trend towards better OS probably related to smaller target volume [34]. Analysis in a retrospective review has not shown any differences in OS after stereotactic or conventionally fractionated re-RT [35]. The similar outcome (mOS of 9.7 months) using conventional-, hypofractionated or SRS techniques was confirmed by another retrospective analysis of re-RT for recurrent malignant glioma [36]. The 9-month mOS with re-RT achieved in our patients with GTV median of 118 cm^3^ falls within the range of previously reported series [17, 25, 32]. There are only very few prospective reports on the efficacy of re-RT. Shi and colleagues recently published the late results from RTOG 0525 trial [37]. Patients received BSC only had an mOS of 4.8 months versus the groups treated with re-RT only, chemotherapy only or radiochemotherapy, 8.2, 10.5, 11.3 months, respectively [37]. It should be noted that in this study OS was calculated from the first progression and not from the beginning of re-RT, as it is in our present study. Well-defined prognostic factors are established for glial tumours; however, the factors influencing the outcome of re-RT are less known. Different factors are considered to influence the efficacy of the survival after re-RT, such as age, performance status, histological grading and the length of the interval between the 1st and the 2nd course of RT [38, 39]. A recent meta-analysis and appraisal summarizes the radiation parameters and outcomes of fractionated re-RT from studies published from 1999 to 2018 [17, 40]. The re-RT was delivered at a median time interval of 12 months (range: 3.5 to 19 months) with dose of 24 to 36 Gy with a daily fractional size of 1.8 to 6 Gy. In our case, > 7 months passed after the 1st RT and we applied 1.6 Gy fraction size. The 8.4 months OS of our group is comparable to previous studies, reporting the mOS from re-RT 7.5 to 11 months.

The evaluation of the clinical data in different series of re-RT revealed important factors, which may improve the survival, such as KPS > 70%, age < 50 years, interval > 12 months between the first RT and re-RT, target volume < 20–30 cm^3^, radiation dose > 30–35 Gy.

In our study, significant predictors for a longer survival after re-RT were the better performance status at re-RT, the longer interval from 1st line treatment to re-RT and lower tumour grade both at diagnosis and at re-RT. The age at re-RT proved not to be a prognostic factor, however, the mean age was below 40 years. The tumour size (i.e. GTV) was one of the most significant factors for the prognosis of our patients, whilst the PTV exhibited no significant relationship to the OS. Recurrent tumour volume remained the strongest factor in multivariate analysis (p = 0.038). The importance of the interval-factor is in line with former reports of re-RT. It can be assumed that the time of the first relapses after the primary treatment is an indicator of the biological behaviour of the tumour [25, 38, 39]. In our patient group, the median survival according to the histopathological grade was higher than in other reported studies (the median survival is around 55–60 months for grade 2 and 18–26 months for grade 3 tumours). [41]. It could be explained with the natural patient selection and the younger age (inclusion of paediatric patients).

In our cases, re-challenge of temosolomide was never applied, hence the primary monotherapy part was not limited in time, it was administered up to progression. Therefore, the MGMT promoter hypermethylation had less importance, because the re-irradiation was delivered when all patients developed resistance to TMZ. The MGMT status defined at the initial diagnosis was available for 27 cases, obviously with no significance on survival after re-RT. Other recent proven biological factors, such as ATRX and IDH- mutation were only partially available in our patient group.

Therefore this report is limited by the lack of detailed molecular analysis as well as by the retrospective methodology which could result in a selection bias as well as an underreporting of low-grade toxicities. However, the selection bias could be reduced by the homogenous treatment concept for our cohort of patients. Nevertheless, comparison to BSC-series remains to be difficult, and conclusions about survival benefits due to intervention should be drawn with caution. Furthermore, due to the still short survival after re-irradiation, objective long-term responses after re-RT were not possible to assess for all patients.

Due to the therapy, amelioration of neurological signs and KPS were experienced in 58% of our patients. Control radiological imaging detected stable disease or partial remission in 44 cases (78.6%).

Although standards of salvage therapy are not yet defined for recurrent glial tumours, mainly due to paucity of high- level prospective or randomized controlled studies, re-RT of various technique is an established salvage option for selected patients [42].

## 5. Conclusion

Smaller recurrent tumour size, better PS, longer interval from 1st line treatment to re-RT and lower tumour grade predict better outcome from re-RT. No radiation-associated serious adverse events were observed and the re-RT improved the performance status and neurologic symptoms in the majority of the cases. Re-irradiation with low fraction size in large volume recurrent gliomas proved to be safe and seems to be clinically beneficial in selected patient group.
